# Migrant Parents and the Psychological Well-Being of Left-Behind Children in Southeast Asia

**DOI:** 10.1111/j.1741-3737.2011.00844.x

**Published:** 2011-08

**Authors:** Elspeth Graham, Lucy P Jordan

**Affiliations:** University of St. Andrews; University of Southampton

**Keywords:** Asian/Pacific Islander families, childhood/children, cross-national, immigration/migrant families, mental health/well-being

## Abstract

Several million children currently live in transnational families, yet little is known about impacts on their health. We investigated the psychological well-being of left-behind children in four Southeast Asian countries. Data were drawn from the CHAMPSEA study. Caregiver reports from the Strengths and Difficulties Questionnaire (SDQ) were used to examine differences among children under age 12 by the migration status of their household (*N* = 3,876). We found no general pattern across the four study countries: Indonesia, the Philippines, Thailand, and Vietnam. Multivariate models showed that children of migrant fathers in Indonesia and Thailand are more likely to have poor psychological well-being, compared to children in nonmigrant households. This finding was not replicated for the Philippines or Vietnam. The paper concludes by arguing for more contextualized understandings.

Transnational migration from the global south is creating new family forms. Growing numbers of parents from low-income countries in Southeast Asia are joining the global movement of workers responding to labor shortages in wealthier countries of the region and beyond. As populations in more developed countries age and demand for service workers grows, an increasing proportion of these migrant parents are mothers who leave their families and children behind to take up “temporary” employment providing domestic and care services to distant others. A common feature of all such migrations is the creation of a transnational family where children are geographically separated from one or both parents over an extended period. It is likely that several million children in the region are currently growing up in the absence of their mother or father, or both, and there is an urgent need for a better understanding of the impacts of family separation on the health and well-being of children left behind. This paper focuses on the psychological well-being of children under 12 years of age in Indonesia, the Philippines, Thailand, and Vietnam using a standardized instrument to measure psychological distress as reported by the child's principal caregiver. Psychological well-being is thus defined as the absence of indicators of psychological distress. The analysis used primary data collected for a reasonably large sample of children in each of the four study countries and is the first study in the region, and to the best of our knowledge worldwide, to explore the psychological well-being of children left behind in low-income countries within an international comparative framework.

This paper brings together two literatures that have developed somewhat independently: first, the international migration literature, which has examined many aspects of the transnational family from a broad social science perspective, and, second, the more specialized literature in child mental health that has focused on the implications of parent–child separation in different contexts, including transnational living arrangements. Whereas the former conceptualizes migration as a household livelihood strategy within the framework of the New Economics of Labor Migration (Stark & Bloom, 1985; Toyota, Yeoh, & Nguyen, 2007), the theoretical foundations of the latter lie in object relations (Ainsworth, Blehar, Waters, & Wall, 1978) and attachment theory (Bowlby, 1958; Grossman, Grossman, & Water, 2005; Lee & Hankin, 2009; Suárez-Orozco, Todorova, & Louie, 2002). The lack of comparable work on the psychological well-being of left-behind children presents particular challenges, as there is no established theoretical framework to guide the analysis. Nevertheless, insights provided by the different emphases and interests within these two literatures make important contributions to understanding the costs and benefits of parental migration for children left behind.

Studies of international labor migration have tended to view the temporary movement of migrants across borders as a family livelihood strategy that aims to improve the socioeconomic circumstances of both the migrant and those left behind. There is now an extensive literature on the impact of remittances sent back by migrants to family members in their countries of origin (e.g., Adams & Page, 2005; Leinbach & Watkins, 1998; Levitt, 2001; Vetrovec, 2004). Although there is some debate about whether labor migration helps to reduce poverty at the macro scale and in the longer term, remittances have been found to improve economic circumstances at the household scale. Those left behind may thus benefit from increases in family income spent on improved nutrition, housing, access to health care, and schooling (Hadi, 1999; Jones & Kittisuksathit, 2003). The strategy is not always successful, however, and debts incurred to facilitate migration or the paucity of remittances may result in left-behind family members having less money than before (Smith-Estelle & Gruskin, 2003).

Fewer studies have examined the social and psychological costs of living in a transnational family. The balance sheet of international labor migration typically involves a trade-off between economic well-being and family proximity. Families divided across national borders may reap economic benefits, but they also make sacrifices in terms of geographical and emotional closeness (Ehrenreich & Hochschild, 2002; Orellana, Thorne, Chee, & Lam, 2001). Such costs may be especially high for mothers separated from their children. The continuing feminization of transnational migration has prompted studies of how gender identities are reworked when women migrate (Elmhirst, 2007; Hondagneu-Sotelo, 1994) and provoked popular anxieties about a care crisis and the future of the family in sending countries such as the Philippines (Asis, Huang, & Yeoh, 2004; Parreñas, 2003). A small but growing body of qualitative work has started to explore emotions, belonging, and intimate relations within transnational families (McKay, 2007; Parreñas, 2001; Svasek, 2008), but with a focus on adults rather than children. The few studies that have examined emotional responses to parental migration among children left behind suggest that children of migrant mothers may be especially prone to anger, feelings of being abandoned or unloved, confusion, and worries (Episcopal Commission for the Pastoral Care of Migrants and Itinerant People–CBCP/Apostleship of the Sea–Manila, Scalabrini Migration Centre & Overseas Workers Welfare Administration [ECMI-CBCP/AOS–Manila, SMC, & OWWA], 2004; Parreñas, 2005). Yet these studies have either relied on qualitative evidence or have been based on limited analysis of quantitative data, and have paid scant attention to the psychological literature on parent–child separation and child mental health.

In mental health studies, separation from a parent has been shown to have detrimental effects on the psychological well-being of children in a number of different circumstances (Amato & Cheadle, 2005; Huurre, Junkkari, & Aro, 2006). Relatively little attention has been paid to separation resulting from migration, and those studies that do examine impacts on children left behind tend to be retrospective (e.g., Parreñas, 2008). The absence of previous work on nonmigrant children in sending households thus leaves a theoretical lacuna, which necessitates a more exploratory approach to analysis. Nevertheless, the negative effects of parent–child separation noted in the wider mental health literature might be expected to arise also in separation resulting from migration. The migration of a parent is a process that transforms family relationships and functioning. Care arrangements for children must be reconfigured, and over time children may form new attachments to “other mothers” (Schmalzbauer, 2004) and change their perceptions of authority figures (Smith, Lalonde, & Johnson, 2004). It is thus plausible to suppose that transnational family arrangements could exact a high emotional cost from both migrant parents and other family members left behind; yet, as Bernhard, Landolt, and Goldring (2005) observe, “[t]here has been a lack of investigation using even the most basic social indicators of well-being and health of children in such situations” (p. 2). The small number of recent studies that have examined children's experience of separation from a parent during the migration process have not only been retrospective but have mostly been conducted in host countries such as the United States or Canada after family reunification. Their findings with respect to child mental health outcomes have been mixed.

Some studies have found no differences between immigrant and nonimmigrant children (Beiser, Hou, Hyman, & Tousignant, 2002), whereas others have reported greater risks for some immigrant groups (Morgan et al., 2007; Suárez-Orozco et al., 2002). Suárez-Orozco and colleagues used data from immigrant youth recently arrived in the United States from various countries of origin to explore separation and reunification among immigrant families. They found that children who arrived as part of a family unit were less likely to report depressive symptoms than children whose families had been geographically separated prior to reunification, but no significant difference between separated and nonseparated children on other measures. The length of separation from a parent was not found to be associated with psychological symptoms. Another study among Caribbean immigrants to Canada, also using standardized scales, found serial migration to be detrimental to parent–child bonding and children's behavior and self-esteem (Smith et al., 2004). Further, it appears that psychological symptoms may manifest later in life. Morgan and colleagues, for example, linked the heightened rate of psychotic disorders among the Caribbean community in London to earlier separation from parents. Other studies have employed qualitative methods, conducting interviews with small samples of immigrant mothers to explore experiences of separation from children (Bernhard et al., 2005) or decisions to separate, as in the case of Chinese mothers in Toronto planning to send their infants back to China (Bohr & Tse, 2009). In these studies, the pain of separation is clearly conveyed by informants, but whether there are measurable effects on the psychological well-being of children, or parents, is not addressed.

## Contexts and Concepts

The literature focusing on immigrant groups in host countries tends to be exploratory in nature and limited in scope because of a lack of adequate data. Most studies adopt the conceptual framework of attachment theory, predicting poor emotional outcomes for those who experience losses or disruptions in primary attachment relationships (Ainsworth et al., 1978; Berlin, Ziv, Amaya-Jackson, & Greenberg, 2007; Bowlby, 1958). Critiques within this literature provide support for two important observations about parent–child separation during serial migration. The first highlights the need for analytical approaches and clinical practice to incorporate cultural diversity, because not all children experiencing transnational family arrangements will react in the same way (Bohr & Tse, 2009). Psychological outcomes may be affected by sociocultural contexts in countries of origin, especially where local social norms favoring extended-family involvement in childrearing challenge models of attachment devised in Euro-American settings (Bernhard et al., 2005; Falicov, 2007; Suárez-Orozco et al., 2002). Second, negative outcomes for the psychological well-being of separated children may vary across different stages in the migration process and over an individual's life course. A limitation of immigrant studies in host countries is that they do not examine child mental health during separation. A reasonable assumption is that retrospective recall after children have experienced the stresses of reunion, including adjustment to a new culture and perhaps separation from a substitute caregiver (Smith et al., 2004), would provide only an indirect indication of well-being during the period of separation from a parent and may actually capture the proximate factors more strongly.

A third observation, well recognized in the migration literature, concerns diversity in the migration process itself. The underlying context in studies conducted among immigrant groups in North America or Europe is the process of serial migration and family reunification in the host country. Although Southeast Asian populations have participated in such migration flows in the past, “temporary” movements of parents to host countries followed by family reunion in the country of origin is now more common. Most left-behind children in the region are not presented with the promise, or threat, of a new life in a foreign land and do not therefore face the same disruptions and losses experienced by immigrant children in Europe or North America. They must still bear the pain of ambiguous loss (Suárez-Orozco et al., 2002) and the uncertainties inherent in maintaining a relationship with a distant parent, but family reunion, if and when it occurs, is likely to take place in familiar surroundings. Whether and in what ways differences in the anticipated location of family reunification influence a child's experience of transnational family arrangements is unknown. Nevertheless, insofar as stability and familiarity during childhood are protective for psychological health, these differences may be significant. Researchers need to develop interdisciplinary theoretical frameworks that link diversity in migration contexts to culturally sensitive understanding of child–parent separation.

## The Psychological Well-Being of Left-Behind Children in Southeast Asia

The prevailing experience of separation from a migrant parent in Southeast Asia is one in which left-behind children expect their absent mother or father to rejoin the family in the country of origin. Often the length of the period of absence is uncertain, as migrants renew short-term (typically 2-year) contracts, perhaps several times, according to family circumstances. Governments in the region promote the temporary out-migration of workers through agreements with foreign governments and recruitment agencies, and in many localities a culture of migration encourages new generations to seek employment abroad. As Asis (2006) notes, in the Philippines wanting to work abroad has become a national obsession. Who goes, where they go, and how long they remain away is thus influenced by multiple factors.

The feminization of transnational labor migration over the past decade has seen the out-migration of more mothers who leave young children behind. This has become a common occurrence in some countries, but not all. In the Philippines and Indonesia, for example, women outnumber men among documented overseas workers, and many are mothers, whereas in Thailand the independent out-migration of married women and mothers is a much rarer event. In the mainly patriarchal societies of the region, social norms regarding the role of women as mothers inform children's expectations of who will nurture them and, consequently, intensify their sense of loss when it is their mother who migrates (Asis, 2006; Parreñas, 2001). This leads us to expect that children of migrant mothers may be at greater risk of poor mental health because most will have experienced separation from their primary caregiver (Suárez-Orozco et al., 2002).

The absence of fathers is understood differently. In her work on fathering from a distance among Filipino transnational families, Parreñas (2008) points out that the migration of Filipino men maintains the traditional gender division of labor and argues that transnational fathering is primarily demonstrated through displays of authority and the imposition of discipline from afar. Gender ideologies are equally influential in promoting public anxieties about the effects of separation on Filipino children. In the Philippines, the migration of mothers has fueled worries about left-behind children becoming spendthrift, delinquent, addicted to drugs, and emotionally scarred (Asis, 2006; ECMI-CBCP/AOS–Manila, SMC, & OWWA, 2004). Yet few studies to date have investigated the potentially different impacts of absent mothers and absent fathers on the psychological well-being of left-behind children, although one early study of 709 Filipino children aged 10 to 12 years concluded that the absence of the mother had the most disruptive effect in terms of lower school grades and poorer social adjustment (Battistella & Conaco, 1998).

The aim of this paper is to extend understanding of the psychological well-being of children in Southeast Asia by investigating whether children in transnational households are more likely to suffer psychological distress than their peers in nonmigrant households. Two hypotheses suggested by the literature on parental absence in the context of transnational labor migration are tested:

Hypothesis 1. Children living in transnational households have poorer psychological well-being (as measured by presence of abnormal emotional symptoms and conduct problems) compared to children living with both parents.

Hypothesis 2. Children of migrant mothers have poorer psychological well-being than children of migrant fathers, when compared to children living with both parents.

Data for each country were analyzed separately because it was anticipated that relationships would vary across different cultural and political settings. Not only were the study samples drawn from different language groups, but the national policy context, which influences the size and composition of transnational migration flows, also varies. Separate analyses for the four study countries allowed a focus on the characteristics of individuals and households within a comparative framework, whereas primary data collection facilitated the comparability of measures.

## Method

Data collected in 2008 as part of a cross-sectional baseline study of Child Health and Migrant Parents in Southeast Asia (CHAMPSEA) were used to examine the relationships between living in a transnational household and child psychological well-being. The CHAMPSEA survey employed a three-stage flexible quota sampling strategy to collect information on about 1,000 target children and their households in each study country—Indonesia, the Philippines, Thailand, and Vietnam. General population sampling was inappropriate in the context of this study, especially given the geographical clustering of high out-migration communities. This, combined with sampling-frame inadequacies, led to the adoption of a sampling process adapted from “sentinel site surveillance” methods, as used in public health studies (Byass et al., 2002). Detailed protocols were developed such that any future replication should produce a sample equivalent in all its major characteristics to the CHAMPSEA sample. Thus the sampling used here is not nationally representative but is based on objective methods suitable to both local circumstances and the objectives of the wider CHAMPSEA project (see Wilson, Huttly, & Fenn, 2006, for a detailed discussion of this approach to sampling).

Stage 1 identified two provinces in each country with rates of international out-migration higher than the national average. In-country experts, using available national migration data combined with local knowledge, selected East Java and West Java in Indonesia, Laguna and Bulacan in the Philippines, Lampang and Udon Thani in Thailand, and Thai Binh and Hai Duong in Vietnam as meeting this criterion. Using local expert knowledge, Stage 2 first identified smaller administrative areas, then local communities (villages in Indonesia and Thailand, barangay in the Philippines, and communes in Vietnam), selecting those with the highest levels of international out-migration as potential study sites. An additional criterion of diversity (either long-established versus more recent out-migration or rural versus more urbanized communities for the Philippines, where migration sites were all long established) governed the final site selection. Stage 3 involved community-based screening surveys to identify and select eligible households and target children. For a household to be eligible for the study, it had to include a child in one of two age groups (3, 4, and 5 years or 9, 10, and 11 years) and fulfill certain criteria in relation to parental migrant status. (The two age groups of pre-school children and children in middle childhood were chosen to focus available resources and ensure adequate observations in each group to support a range of analyses.) The sample excluded single-parent households. Qualifying households were those in which either (a) both parents had been usually resident at the same address as the target child for a period of at least 6 months prior to interview or (b) one or both parents had been working overseas for a continuous period of at least 6 months prior to interview and neither parent was an internal migrant. As the aim was to oversample transnational households in order to fulfill the study's objectives, quotas specified that at least half of the households selected should belong to the second category, with a balance, where appropriate, between migrant father households and migrant mother households. Screening proceeded from more than one location in each community to avoid spatial clustering biases, and quotas were filled systematically. Only one target child was identified in each household, and quotas ensured approximately equal numbers of girls and boys and young and older children. If more than one child was eligible, supervisors were instructed to randomly select one of the children and assign this child for recruitment to the interviewer. For each province, eight quotas defined by household migration status, child age, and child gender were then filled systematically according to which cell in the sampling matrix was most under quota. As each cell began to fill up, field workers encountered a few households where two children of the same age and gender both qualified for the same sub-quota. In this instance, a child who was available and amenable was recruited, which may have biased the sample slightly. Across the four countries, however, the number of households with same gender–age eligibility was low (6.9% across the full sample and the entire data collection period). Qualifying households agreeing to participate were recruited to the study, and screening proceeded until all specified quotas had been filled. Though the samples are not nationally representative, they are of sufficient size to conduct comparative analyses. For ease of reporting, the country name is used here when referring to the country samples.

In each household recruited to the survey, interviews were conducted in local languages with a responsible adult, the primary caregiver of the target child and, for the older age group, the target child. Data for the present study were drawn from the responsible adult and primary caregiver interviews. Questionnaires were compiled in English, and translation/back-translation used to ensure that meanings in local versions were as near as possible to the original. Translation for standardized measures, including the Strengths and Difficulties Questionnaire (SDQ), followed a more rigorous protocol. The protocol for the SDQ was based on other international studies (Hunt & Bhopal, 2004) and devised in conjunction with Robert Goodman, who holds the copyright for the SDQ. The protocol included two separate translations and back-translations and the convening of a committee to discuss and resolve any disparities. We were given permission to use approved versions of the SDQ in Vietnamese, Thai, and Bahasa. The three questions that differ on the version for children aged 3 and 4, however, had to be translated for each of these three countries. In addition, the impact assessment had to be translated into Bahasa Indonesian. The full version had not been completed for the Philippines, and therefore the SDQ in its entirety was translated for this country. Cognitive questions in the pilot surveys were used to test local understandings of particular questions, and translations were revised where problems occurred. Experienced interviewers fluent in local languages were recruited in each study country and given standard training. The purpose of the study was explained to informants, confidentiality assured, and verbal informed consent obtained prior to interview. In administering the survey, we sought to ensure that data collected were comparable across the four study countries. Ethics approval was obtained from the National University of Singapore, University of St. Andrews, Scalabrini Migration Center (Philippines), Center for Population and Policy Studies, Gadjah Mada University (Indonesia), Institute for Population and Social Research, Mahidol University (Thailand), and Asia-Pacific Economic Center (Vietnam).

### Child Psychological Well-Being

Children living with both parents in the same communities as children living in transnational households provided a country-specific benchmark for comparison. Some children had both parents working abroad (*n* = 185). These cases were dropped from the current analysis in order to examine differences by gender of migrant parent, as were 10 cases where data were problematic or missing. In addition, three cases of children in mother-migrant transnational households were excluded from the sample for Thailand. Despite considerable efforts, very few households where Thai mothers had migrated, leaving behind children under 12 years of age, were found. Table 1 shows the breakdown of the final sample by country and age of child (*N* = 3,876).

**Table 1:** Composition of Sample (*N* = 3,876)

	Country							
	
	Indonesia		Philippines		Thailand		Vietnam	
				
Migrant Status of Household	Young Child	Older Child	Young Child	Older Child	Young Child	Older Child	Young Child	Older Child
Parents usually resident	250	246	245	246	255	257	230	231
Father current migrant	94	79	204	171	237	246	127	87
Mother current migrant	141	151	27	65	—	—	118	169
Column total	485	476	476	482	492	503	475	487
Country total	961		958		995		962	

*Note:* Young child = 3, 4, or 5 years of age; older child = 9, 10, or 11 years of age.

Poor psychological well-being was interpreted as the presence of certain symptoms and behaviors indicative of a mental disorder and thus of psychological distress. Possible cases of mental disorder were identified using the 25-item SDQ, a screening tool developed by Robert Goodman in the United Kingdom (R. Goodman, 1997). The SDQ is now available in more than 60 languages and is widely employed in Europe and North America. There have been fewer studies reporting on its use in low-income countries, although it has been used in Asia and the Middle East (Fuhr & De Silva, 2008; R. Goodman, Renfrew, & Mullick, 2000; Samad, Hollis, Prince, & Goodman, 2005; Woerner et al., 2004).

The 25 core items of the SDQ were completed by the child's primary caregiver, and five subscales, each of five items, were derived, along with the Total Difficulties score. Each subscale can be viewed as a distinct measure allowing investigation of specific aspects of child mental health (Palmieri & Smith, 2007). This analysis focuses on two subscales that screen for emotional symptoms and conduct problems, an internalizing and an externalizing subscale, respectively. These subscales were preferred to the Total Difficulties scale, which has been found to be less reliable in a non-Western context (Mullick & Goodman, 2001). Gender differences in externalizing and internalizing problems are well known across different cultural contexts as illustrated by a 12-country comparative study using the Child Behavior Checklist (CBCL; Crinjnen, Achenbach, & Verhulst, 1997). Research has also contributed to understanding the important role that conduct-type behavioral disorders play during the preschool years (Egger & Angold, 2006). Further, both the dimensions of emotion and conduct are particularly relevant to left-behind children of migrants, who are predicted to experience emotional problems following parent–child separation, especially if the separation is from the mother. Past work also suggests more conduct problems among children of migrant fathers when mothers left behind struggle to cope with the traditionally masculine role of disciplining their offspring (Battistella & Conaco, 1998; Hugo, 2002).

All analyses used a dichotomous measure distinguishing normal/borderline cases from cases in the abnormal range. Given that the SDQ is a screening tool for detecting likely mental health problems, we took the conservative view that only scores within the abnormal range should be treated as possible cases of mental disorder. In the absence of normative data for our study countries, estimates of the frequency of possible mental disorders were based on cut-offs developed by Goodman for the United Kingdom. This strategy has been employed previously in the Asian context in Bangladesh (R. Goodman et al., 2000), Pakistan (Samad et al., 2005), and Sri Lanka (Prior, Virasinghe, & Smart, 2005). Scores for the two 5-item subscales of interest range between 0 and 10, with scores >4 for Emotional Symptoms and >3 for Conduct Problems predicting cases of mental disorder. The psychometric properties of the SDQ have been validated for clinical and community samples in other low-income and Asian settings (Du, Kou, & Coghill, 2008; R. Goodman et al., 2000; Matsuishi et al., 2008; Mullick & Goodman, 2001; Syed, Hussein, & Mah, 2007).

### Measurement of Covariates

We hypothesized first that the frequency of predicted abnormal scores on emotional symptoms and conduct problems would be higher among children living in transnational households, compared with those living with both parents in nonmigrant households. Second, following previous studies, we hypothesized differences among children in transnational households, such that the frequency of predicted emotional and conduct disorders would be highest among children of migrant mothers. Three types of transnational household were distinguished according to which parent was absent and whether the principal caregiver of the child was the left-behind mother (father-migrant/ mother-caregiver), the left-behind father (mother-migrant/father-caregiver), or “other” (parent-migrant/other-caregiver, a mixed group but mainly grandparent caregivers in mother-migrant households). The second of these categories was dropped for Thailand. The group of children in nonmigrant households, where both parents were usually resident, was used as the reference category in all models. Table 2 presents the distribution of cases with abnormal scores (i.e., scores above the cut point) on emotional symptoms and on conduct problems, by migrant/caregiver status, child age, and child gender across the four study countries. There are noticeable differences in the overall percentage of abnormal scores between the countries, which may reflect variability in culturally acceptable child behaviors.

**Table 2:** Percentage of Abnormal Scores on Child Psychological Well-Being Measures by Migration Status, Child Gender, and Child Age

	Indonesia	Philippines	Thailand	Vietnam
Emotional symptoms				
Overall	30.28	22.65	11.26	24.43
Parent migration & caregiver				
Parents usually resident	25.4	25.66	11.33	24.95
Father-migrant/mother-caregiver	42.51	18.9	11.09	33.52
Mother-migrant/father-caregiver	31.66	16.36	—	15.2
Parent-migrant/other-caregiver	31.31	25	20	24.58
Child gender				
Boy	31.82	18.79	9.98	19.91
Girl	28.72	26.51	12.55	28.69
Child age				
Young child	33.61	20.59	10.16	23.16
Older child	26.89	24.69	12.33	25.67
Conduct Problems				
Overall	21.64	25.89	27.14	9.15
Parent migration & caregiver				
Parents usually resident	21.77	29.12	24.22	10.85
Father-migrant/mother-caregiver	27.54	22.67	30.33	8.38
Mother-migrant/father-caregiver	15.58	10.91	—	6.86
Parent-migrant/other-caregiver	23.23	30.88	20	7.63
Child gender				
Boy	24.17	27.56	30.34	13.7
Girl	19.08	24.22	23.89	4.85
Child age				
Young child	37.73	37.39	40.65	11.58
Older child	5.25	14.52	13.92	6.78

*Note:* Young child = 3, 4, or 5 years of age. Older child = 9, 10, or 11 years of age.

For the father-migrant/mother-caregiver and the mother-migrant/father-caregiver groups, the gender of the person reporting SDQ scores for the target child is known, as it was the indicated caregiver in all cases. For children in the reference group, SDQ scores were reported mainly by mothers (91% in Indonesia, 92% in the Philippines, 94% in Thailand, and 83% in Vietnam). A minority of SDQ scores for these children in “usually resident” households were reported by fathers or other members of the household, such as grandparents. Possibly, generational and gender differences in the reporter could influence the observed outcomes, although prior research suggested that grandparents, for example, reliably reported child mental health problems (Palmieri & Smith, 2007).

As this is the first study of its kind, a central concern was to establish whether or not there was a significant association between child psychological well-being and (a) the migration status and (b) the migration/caregiver status of the household in which the child lived. In this respect, the models reported in Tables 3–5 are exploratory rather than explanatory and include covariates identified from the relevant literature on child mental health rather than factors (e.g., frequency of contact with migrant parent) that might “explain” outcomes for children in different types of household within each country. Only those factors identified as potential confounders of the relationship between the key predictor of household migration/caregiver status and emotion and conduct disorders were therefore included in the multivariate models. These relate to characteristics of the child, the caregiver, and the household. First, SDQ ratings for community samples have been found to vary according to the age and gender of the child (Du et al., 2008; Matsuishi et al., 2008; Prior et al., 2005). Therefore, child age (3–5 years vs. 9–11 years) and child gender were controlled in all models. In addition, the parity of the child was included to reflect position in the household and, for those countries with sufficient (though small) numbers, child long-term disability to account for mental disorders that may be related to comorbidity (Fuhr & De Silva, 2008) rather than parental migration.

**Table 3:** Summarized Multivariate Models for Child Emotional Symptoms and Conduct Problems

Emotional Symptoms																

	Indonesia (*n* = 961)								Vietnam (*n* = 962)							
		
	Model A				Model B				Model A				Model B			
				
	*β*	*SE*	*eβ*	95% CI	*β*	*SE*	*eβ*	95% CI	*β*	*SE*	*eβ*	95% CI	*β*	*SE*	*eβ*	95% CI
Parents usually resident			1.00				1.00				1.00				1.00	
Transnational household	0.48**	0.14	1.62	[1.22, 2.14]	0.45	0.16	1.57	[1.15, 2.13]	−0.06	0.15	0.94	[0.70, 1.26]	−0.09	0.16	0.91	[0.67, 1.24]
Child gender																
Boy																
Girl	−0.15	0.14	0.86	[0.65, 1.14]	−0.13**	0.15	0.88	[0.65, 1.18]	0.48**	0.15	1.61	[1.20, 2.18]	0.47**	0.16	1.59	[1.17, 2.16]
Child age																
Young																
Older	−0.32*	0.14	0.72	[0.55, 0.96]	−0.46	0.16	0.63	[0.46, 0.87]	0.12	0.15	1.13	[0.84, 1.52]	0.02	0.16	1.02	[0.74, 1.40]
Log likelihood	−580.37				−528.52				−529.38				−518.45			

	Philippines (*n* = 958)								Thailand (*n* = 995)							
		
	Model A				Model B				Model A				Model B			
				
	*β*	*SE*	*eβ*	95% CI	*β*	*SE*	*eβ*	95% CI	*β*	*SE*	*eβ*	95% CI	*β*	*SE*	*eβ*	95% CI
Parents usually resident			1.00				1.00				1.00				1.00	
Transnational household	−0.35*	0.16	0.70	[0.52, 0.96]	−0.07	0.18	0.93	[0.66, 1.33]	−0.01	0.20	0.99	[0.67, 1.46]	−0.06	0.21	0.94	[0.63, 1.42]
Child gender																
Boy																
Girl	0.44**	0.16	1.56	[1.14, 2.12]	0.46**	0.16	1.58	[1.14, 2.18]	0.25	0.20	1.29	[0.87, 1.91]	0.22	0.22	1.24	[0.83, 1.87]
Child age																
Young																
Older	.24	0.16	1.27	[0.94, 1.73]	.23	0.17	1.26	[0.90, 1.76]	.21	0.20	1.24	[0.83, 1.84]	.17	0.21	1.18	[0.77, 1.81]
Log likelihood	−504.75				−471.18				−348.70				−325.22			

Conduct Problems																

	Indonesia (*n* = 961)								Vietnam (*n* = 962)							
		
	Model A				Model B				Model A				Model B			
				
	*β*	*SE*	*eβ*	95% CI	*β*	*SE*	*eβ*	95% CI	*β*	*SE*	*eβ*	95% CI	*β*	*SE*	*eβ*	95% CI
Parents usually resident			1.00				1.00				1.00				1.00	
Transnational household	−0.03	0.17	0.97	[0.70, 1.36]	−0.07	0.18	0.93	[0.66, 1.32]	−0.39	0.23	0.68	[0.43, 1.06]	−0.36	0.24	0.69	[0.44, 1.11]
Child gender																
Boy																
Girl	−0.38*	0.17	0.68	[0.49, 0.96]	−0.38*	0.17	0.69	[0.49, 0.97]	−1.13***	0.25	0.32	[0.20, 0.53]	−1.17***	0.25	0.31	[0.19, 0.51]
Child age																
Young																
Older	−2.41***	0.23	0.09	[0.06, 0.14]	−2.31***	0.24	0.10	[0.06, 0.16]	−0.57*	0.23	0.57	[0.36, 0.90]	−0.69*	0.25	0.50	[0.31, 0.82]
Log likelihood	−416.96				−409.59				−278.14				−272.90			

	Philippines (*n* = 958)								Thailand (*n* = 995)							
		
	Model A				Model B				Model A				Model B			
				
	*β*	*SE*	*eβ*	95% CI	*β*	*SE*	*eβ*	95% CI	*β*	*SE*	*eβ*	95% CI	*β*	*SE*	*eβ*	95% CI
Parents usually resident			1.00				1.00				1.00				1.00	
Transnational household	−0.37*	0.15	0.69	[0.51, 0.93]	−0.32	0.17	0.73	[0.52, 1.02]	0.34*	0.15	1.41	[1.05, 1.89]	0.38*	0.15	1.46	[1.08, 1.98]
Child gender																
Boy																
Girl	−0.20	0.15	0.82	[0.60, 1.11]	−0.23	0.16	0.79	[0.58, 1.08]	−0.32*	0.15	0.70	[0.54, 0.94]	−0.36*	0.15	0.70	[0.52, 0.94]
Child age																
Young																
Older	−1.27***	0.16	0.28	[0.21, 0.39]	−1.23***	0.17	0.29	[0.21, 0.41]	−1.45***	0.16	0.23	[0.17, 0.32]	−1.47***	0.17	0.23	[0.17, 0.32]
Log likelihood	−510.63				−497.33				−530.34				−514.97			

*Note:* All models labeled “B” contain the same variables as Models “D” in Tables 4 and 5. Young = ages 3, 4, or 5. Older = ages 9, 10, or 11. CI = confidence interval.

* *p* < .05.

** *p* < .01.

*** *p* < .001.

**Table 4:** Multivariate Models Predicting Child Emotional Symptoms in Four Southeast Asian Countries

	Indonesia (*n* = 961)								Philippines (*n* = 958)							
		
	Model C				Model D				Model C				Model D			
				
	*β*	*SE*	*eβ*	95% CI	*β*	*SE*	*eβ*	95% CI	*β*	*SE*	*eβ*	95% CI	*β*	*SE*	*eβ*	95% CI
Parents usually resident			1.00				1.00				1.00				1.00	
Father-migrant/mother-caregiver	0.77***	0.19	2.16	[1.50, 3.13]	0.96***	0.21	2.60	[1.72, 3.94]	−0.38*	0.17	0.69	[0.49, 0.96]	−0.09	0.20	0.91	[0.62, 1.34]
Mother-migrant/father-caregiver	0.33	0.19	1.39	[0.97, 2.00]	0.28	0.20	1.33	[0.89, 1.97]	−0.60	0.38	0.55	[0.26, 1.16]	−0.29	0.41	0.75	[0.34, 1.66]
Parent-migrant/other-caregiver	0.26	0.24	1.30	[0.81, 2.08]	−0.10	0.27	0.91	[0.54, 1.54]	−0.07	0.30	0.93	[0.52, 1.67]	0.17	0.33	1.19	[0.63, 2.27]
Child gender																
Boy																
Girl	−0.15	0.14	0.86	[0.65, 1.13]	−0.13	0.15	0.88	[0.65, 1.19]	0.43**	0.16	1.54	[1.13, 2.09]	0.45**	0.16	1.57	[1.13, 2.16]
Child age																
Young																
Older	−0.32*	0.14	0.73	[0.55, 0.96]	−0.47**	0.17	0.62	[0.45, 0.86]	0.25	0.16	1.29	[0.95, 1.75]	0.24	0.17	1.27	[0.90, 1.78]
Child parity																
Parity 1							1.00								1.00	
Parity 2					−0.01	0.18	0.99	[0.70, 1.40]					−0.11	0.21	0.90	[0.60, 1.34]
Parity 3 or more					−0.18	0.21	0.83	[0.55, 1.26]					−0.04	0.21	0.96	[0.63, 1.45]
Child long-term disability																
No							1.00								1.00	
Yes					0.26	0.88	1.29	[0.23, 7.23]					0.56	0.53	1.75	[0.61, 4.98]
Caregiver education																
Primary or less						1.00								1.00		
Lower secondary					−0.08	0.20	0.92	[0.62, 1.37]					−0.50	0.44	0.60	[0.26, 1.43]
Upper secondary or more					−0.55*	0.24	0.58	[0.36, 0.93]					−0.21	0.20	0.81	[0.55, 1.19]
Caregiver mental health problem																
No							1.00								1.00	
Yes					1.40***	0.16	4.04	[2.93, 5.57]					1.12***	0.21	3.08	[2.06, 4.61]
Household wealth																
Low							1.00								1.00	
Medium					−0.19	0.19	0.82	[0.57, 1.19]					−0.64**	0.22	0.53	[0.34, 0.82]
High					−0.55*	0.22	0.58	[0.38, 0.88]					−1.02***	0.24	0.36	[0.23, 0.57]
Younger sibling/s																
No							1.00								1.00	
Yes					0.31	0.18	1.36	[0.95, 1.96]					0.01	0.18	1.01	[0.71, 1.44]
Log likelihood	−577.66				−521.63				−504.03				−470.69			

	Thailand (*n* = 995)								Vietnam (*n* = 962)							
		
	Model C				Model D				Model C				Model D			
				
	*β*	*SE*	*eβ*	95% CI	*β*	*SE*	*eβ*	95% CI	*β*	*SE*	*eβ*	95% CI	*β*	*SE*	*eβ*	95% CI
Parents usually resident			1.00				1.00				1.00				1.00	
Father-migrant/mother-caregiver	−0.02	0.20	0.98	[0.66, 1.45]	−0.06	0.21	0.94	[0.62, 1.42]	0.43*	0.19	1.53	[1.05, 2.24]	0.34	0.20	1.40	[0.95, 2.07]
Mother-migrant/father-caregiver	—	—	—		—	—	—		−0.65**	0.23	0.52	[0.34, 0.81]	−0.67**	0.23	0.51	[0.32, 0.81]
Parent-migrant/other-caregiver	0.78	1.13	2.18	[0.24, 19.98]	0.23	1.21	1.26	[0.12, 13.50]	−0.02	0.24	0.98	[0.61, 1.58]	−0.02	0.26	0.98	[0.59, 1.65]
Child gender																
Boy																
Girl	0.26	0.20	1.30	[0.87, 1.93]	0.22	0.21	1.25	[0.83, 1.87]	0.47**	0.15	1.60	[1.18, 2.16]	0.46**	0.16	1.58	[1.16, 2.15]
Child age																
Young																
Older	0.22	0.20	1.24	[0.83, 1.84]	0.17	0.22	1.26	[0.77, 1.82]	0.22	0.15	1.25	[0.92, 1.69]	0.15	0.17	1.16	[0.84, 1.60]
Child parity																
Parity 1							1.00								1.00	
Parity 2					−0.59*	0.24	0.55	[0.35, 0.88]					−0.20	0.19	0.82	[0.57, 1.20]
Parity 3 or more					−0.71	0.40	0.49	[0.22, 1.08]					0.06	0.28	1.06	[0.62, 1.82]
Child long-term disability																
No							1.00								1.00	
Yes					1.98	1.12	7.22	[0.80, 64.96]					−0.73	0.79	0.48	[0.10, 2.26]
Caregiver education																
Primary or less							1.00								1.00	
Lower secondary					0.06	0.27	1.06	[0.62, 1.81]					0.13	0.21	1.13	[0.75, 1.73]
Upper secondary or more					−0.30	0.31	0.74	[0.40, 1.36]					−0.19	0.28	0.83	[0.48, 1.44]
Caregiver mental health problem																
No							1.00								1.00	
Yes					1.27***	0.22	3.57	[2.33, 5.48]					0.52**	0.20	1.68	[1.14, 2.48]
Household wealth																
Low							1.00								1.00	
Medium					−0.10	0.25	0.91	[0.55, 1.49]					0.46*	0.18	1.58	[1.11, 2.25]
High					0.41	0.27	1.51	[0.88, 2.58]					0.51*	0.21	1.66	[1.11, 2.50]
Younger sibling/s																
No							1.00								1.00	
Yes					−0.49	0.29	0.61	[0.35, 1.07]					0.11	0.20	1.11	[0.75, 1.65]
Log likelihood	−348.48				−325.19				−520.01				−510.91			

*Note: Young* = age 3, 4, or 5; Older = age 9, 10, or 11. CI = confidence interval.

* *p* < 0.05.

** *p* < 0.01.

*** *p* < 0.001.

**Table 5:** Multivariate Models Predicting Child Conduct Problems in Four Southeast Asian Countries

	Indonesia (*n* = 961)								Philippines (*n* = 958)							
		
	Model C				Model D				Model C				Model D			
				
	*β*	*SE*	*eβ*	95% CI	*β*	*SE*	*eβ*	95% CI	*β*	*SE*	*eβ*	95% CI	*β*	*SE*	*eβ*	95% CI
Parents usually resident			1.00			1.00				1.00				1.00		
Father-migrant/mother-caregiver	0.31	0.23	1.37	[0.88, 2.13]	0.19	0.24	1.21	[0.76, 1.92]	−0.41*	0.17	0.66	[0.48, 0.92]	−0.35	0.18	0.71	[0.49, 1.02]
Mother-migrant/father-caregiver	−0.30	0.24	0.74	[0.46, 1.19]	−0.33	0.25	0.72	[0.44, 1.17]	−0.95*	0.46	0.38	[0.16, 0.94]	−0.92*	0.47	0.40	[0.16, 1.00]
Parent-migrant/other-caregiver	−0.16	0.28	0.85	[0.49, 1.48]	−0.13	0.30	0.88	[0.49, 1.58]	0.11	0.29	1.12	[0.63, 1.99]	0.11	0.31	1.12	[0.61, 2.05]
Child gender																
Boy							1.00								1.00	
Girl	−0.39*	0.17	0.68	[0.48, 0.95]	−0.38*	0.18	0.68	[0.48, 0.96]	−0.23	0.16	0.79	[0.59, 1.08]	−0.26	0.16	0.77	[0.57, 1.06]
Child age																
Young							1.00								1.00	
Older	−2.40***	0.23	0.09	[0.06, 0.14]	−2.30***	0.24	0.10	[0.06, 0.16]	−1.25***	0.16	0.29	[0.21, 0.39]	−1.21***	0.17	0.30	[0.21, 0.42]
Child parity																
Parity 1							1.00								1.00	
Parity 2					0.05	0.20	1.05	[0.72, 1.55]					−0.18	0.19	0.83	[0.57, 1.21]
Parity 3 or more					−0.30	0.26	0.74	[0.45, 1.23]					−0.49*	0.21	0.61	[0.41, 0.91]
Child long-term disability																
No															1.00	
Yes					—	—	—						0.77	0.56	2.17	[0.73, 6.44]
Caregiver education																
Primary or less							1.00								1.00	
Lower secondary					0.41	0.23	1.51	[0.97, 2.35]					−0.04	0.43	0.96	[0.41, 2.22]
Upper secondary or more					0.19	0.25	1.21	[0.74, 1.98]					−0.13	0.20	0.88	[0.60, 1.31]
Caregiver mental health problem																
No							1.00								1.00	
Yes					0.47*	0.20	1.61	[1.09, 2.36]					0.71**	0.22	2.04	[1.33, 3.11]
Household wealth																
Low							1.00								1.00	
Medium					0.14	0.23	1.15	[0.74, 1.79]					−0.14	0.23	0.87	[0.55, 1.35]
High					0.13	0.25	1.14	[0.70, 1.85]					−0.41	0.24	0.66	[0.42, 1.05]
Younger sibling(s)																
No							1.00								1.00	
Yes					−0.23	0.24	0.79	[0.49, 1.27]					−0.36*	0.17	0.70	[0.50, 0.98]
Log likelihood	−414.43				−407.97				−508.12				−495.24			

	Thailand (*n* = 995)								Vietnam (*n* = 962)							
		
	Model C				Model D				Model C				Model D			
				
	*β*	*SE*	*eβ*	95% CI	*β*	*SE*	*eβ*	95% CI	*β*	*SE*	*eβ*	95% CI	*β*	*SE*	*eβ*	95% CI
Parents usually resident			1.00				1.00				1.00				1.00	
Father-migrant/mother-caregiver	0.35*	0.15	1.42	[1.05, 1.91]	0.39*	0.16	1.48	[1.09, 2.01]	−0.30	0.31	0.74	[0.40, 1.36]	−0.34	0.32	0.72	[0.38, 1.34]
Mother-migrant/father-caregiver	—	—	—		—	—	—		−0.45	0.32	0.64	[0.34, 1.19]	−0.40	0.33	0.67	[0.35, 1.29]
Parent-migrant/other-caregiver	−0.48	1.16	0.62	[0.06, 5.95]	−0.87	1.20	0.42	[0.04, 4.35]	−0.41	0.38	0.66	[0.31, 1.41]	−0.35	0.42	0.70	[0.31, 1.59]
Child gender																
Boy			1.00								1.00					
Girl	−0.33*	0.15	0.721	[0.54, 0.97]	−0.37*	0.15	0.69	[0.51, 0.94]	−1.13***	0.25	0.32	[0.20, 0.53]	−1.17***	0.25	0.31	[0.19, 0.51]
Child age																
Young			1.00								1.00					
Older	−1.46***	0.16	0.233	[0.17, 0.32]	−1.48***	0.17	0.23	[0.16, 0.32]	−0.56*	0.24	0.57	[0.36, 0.91]	−0.68**	0.25	0.51	[0.31, 0.83]
Child parity																
Parity 1							1.00								1.00	
Parity 2					−0.28	0.18	0.75	[0.53, 1.07]					0.01	0.29	1.01	[0.57, 1.77]
Parity 3 or more					−0.20	0.28	0.81	[0.48, 1.40]					0.31	0.39	1.37	[0.64, 2.95]
Child long-term disability																
No							1.00								1.00	
Yes					0.88	1.17	2.41	[0.24, 24.07]					—	—	—	
Caregiver education																
Primary or less							1.00								1.00	
Lower secondary					0.04	0.20	1.04	[0.70, 1.55]					0.03	0.31	1.03	[0.56, 1.89]
Upper secondary or more					0.37	0.22	1.44	[0.94, 2.23]					−0.47	0.43	0.62	[0.27, 1.46]
Caregiver mental health problem																
No							1.00								1.00	
Yes					0.71***	0.18	2.04	[1.42, 2.93]					0.63*	0.28	1.88	[1.09, 3.26]
Household wealth																
Low							1.00								1.00	
Medium					−0.21	0.18	0.81	[0.57, 1.14]					−0.22	0.27	0.80	[0.47, 1.37]
High					−0.76**	0.23	0.47	[0.30, 0.73]					0.17	0.31	1.19	[0.65, 2.16]
Younger sibling(s)																
No							1.00								1.00	
Yes					−0.33	0.22	0.72	[0.47, 1.10]					0.09	0.32	1.09	[0.58, 2.04]
Log likelihood	−530.04				−514.32				−278.06				−272.88			

*Note:* Young = ages 3, 4, or 5. Older = ages 9, 10, or 11. CI = confidence interval.

* *p* < .05.

** *p* < .01.

*** *p* < .001.

The second group of potential confounders concerns characteristics of the principal caregiver who completed the SDQ for the child in his or her care. As a tendency for depressed mothers to overreport problem behaviors in their children has been reported by Davé, Nazareth, Senior, and Sherr (2008), a measure of caregiver mental health derived from the 20-item Self-Reporting Questionnaire (SRQ–20) was included. The SRQ–20 is recommended by the World Health Organization and widely used to screen for mental health problems. It has been validated for Vietnam, along with many other countries (Tuan, Harpham, & Huong, 2004). The suggested cut off point of 7/8, with scores of 8 or more defining “cases,” was used to identify probable mental health problems. The highest educational level achieved by caregivers was also added, as this may influence their understanding of and report on the behavior of the child in their care.

Household characteristics are likely to act as confounders in the relationship between household migration status (or migration/caregiver status) and child mental health. Socioeconomic status is known to be associated with the incidence of mental health problems in children in low-income as well as high-income countries (Hackett, Hackett, Bhakta, & Gowers, 1999; Prior et al., 2005). In the current study, socioeconomic status was measured by a household wealth index based on a methodology developed for the Young Lives project (see http://www.younglives.org.uk). The index averages scores for housing quality, consumer durables, and basic amenities and has recently been used in a study of maternal mental health in four low-income countries (De Silva, Huttly, Harpam, & Kenward, 2007). For each of the CHAMPSEA study countries, households were grouped into three relative-wealth categories according to their average scores. In addition, presence of siblings in the household was considered. As Smith and colleagues (2004) noted, having older siblings may be a protective factor, whereas the presence of younger siblings may increase stress for a child charged with their care. Thus, to complement child parity, which is an indicator of the number of older siblings, the absence/presence of younger siblings was added. Together these two variables provide a summary of numbers of children and the target child's position in the family.

### Statistical Methods

Two sets of multivariate logistic regression models were fitted. The first set (Table 3, Models A and B) modeled outcomes for two groups of children by household migration status (nonmigrant vs. transnational households) on the two SDQ subscales (emotional symptoms and conduct problems, respectively). The second set (Tables 4 and 5, Models C and D) used a subdivision of transnational households to investigate the associations between migration/caregiver status and emotional and conduct disorders. In both sets, basic models were fitted first for each of the four countries and for the two mental health measures, accounting only for child age and child gender. Next, the group of other possible confounders was added to each model in order to ascertain whether any relationship between household migration status (or migration/caregiver status) and child mental disorders remained once known covariates were taken into account. The same models were fitted for each country, with the exception of the exclusion of child long-term disability due to small numbers in the extended models for conduct problems in Indonesia and Vietnam. Interactions between migration/caregiver status and other household structure variables and between child parity and younger siblings were investigated, but no significant and stable interactions that improved model fit were found (results not shown). In the absence of an established theoretical basis for their inclusion, interactions were dropped from the final model on the grounds of parsimony. Beta coefficients, standard errors, and exponentiated beta coefficients are reported in Tables 3, 4, and 5.

In all models, children in transnational households were compared to children in nonmigrant households with both parents “usually resident.” The predominance of mother-caregivers reporting SDQ scores for children in this reference group has been noted. To investigate whether the presence of a minority of other-caregivers reporting SDQ scores impacts on model results, these cases were dropped and all multivariate models were rerun. The results (not shown) confirmed the stability of the original models. With few exceptions, the significant associations found in the extended models (Models B and D) were the same as those in the original models.

## Results

### Children in Transnational Households Versus Children Living With Both Parents

Table 3 presents a summary of the first set of models (Models A and B) examining differences between the group of children living in transnational households where one parent is absent and those living with both parents. Model A includes child age and child gender, and Model B adds the additional confounders. The structure of both models is identical to that of Models C and D, respectively, with the exception of the migration status variable. To avoid repetition, comments are confined to the results presented in Table 3 as they relate to Hypothesis 1. The detailed results are presented below in relation to Hypothesis 2.

The relationship between living in a transnational household and experiencing emotional problems differed across the four countries. Once child age (younger and older age groups) and gender were accounted for in the basic model predicting emotional symptoms (Table 3, Emotional Problems, Model A for each country), significant differences between children in the two types of household were found only for Indonesia and the Philippines, and then with a relationship in opposite directions. Whereas left-behind children in transnational households in Indonesia were more likely to suffer emotional distress compared to children living with both parents (*e*^*β*^ = 1.62;*β* = .48), the opposite appeared to be the case in the Philippines (*e*^*β*^ = 0.70;*β* = −.35). When the other identified confounders were added to these models (Table 3, Emotional Problems, Model B for each country), however, only the relationship for Indonesia remained significant, with a slight downward trend in the point estimates (*e*^*β*^ = 1.57;*β* = .45), lending support to Hypothesis 1. No significant differences between children living with both parents and children living in transnational households were found for either Thailand or Vietnam.

The models for conduct problems are also summarized in Table 3. Only for Thailand did the results for conduct problems produce some support for Hypothesis 1, with increased odds of children in transnational households experiencing conduct problems compared to children living with both parents in both the basic (*e*^*β*^ = 1.41;*β* = .34) and the extended (*e*^*β*^ = 1.46;*β* = .38) models (Table 3, Conduct Problems, Models A and B respectively for Thailand). Indeed the addition of all the identified confounders strengthened the relationship slightly. No significant differences were found between the two groups of children in either Indonesia or Vietnam. In the Philippines the difference was significant in the basic model but not in the anticipated direction. Thus left-behind Filipino children may be *less* likely to have conduct problems compared to their counterparts living with both parents, although the relationship does not quite reach significance in the extended model (*p* < .10). No country showed the hypothesized difference on both subscales, and results for the Philippines sample indicate that transnational family arrangements may not be harmful to the psychological well-being of some children.

Only for Indonesia in relation to emotional symptoms and Thailand in relation to conduct problems were children in transnational households found to be clearly worse off than children in nonmigrant households. Results for the Philippines and Vietnam support rejection of Hypothesis 1, which anticipates a universal relationship. Country-specific variations raise the possibility that the association between the migration status of the household and child psychological well-being is culturally contextualized. Perhaps what is relatively detrimental to children in one set of circumstances is relatively advantageous in another. Nevertheless, these findings possibly also reflect within-country differences or the heterogeneous nature of the transnational household group, or both. Further analysis, incorporating a more detailed categorization of transnational households therefore examined multivariate associations to test the second hypothesis, which expects differences related to the gender of the migrant parent (and hence the child's main caregiver).

### Children in Different Types of Transnational Households Versus Children Living With Both Parents

In order to test Hypothesis 2, the category of transnational households was subdivided, and a composite variable was derived incorporating both the gender of the migrant parent and the gender of child's main caregiver (migrant/caregiver status). Three categories were identified: (a) children with a migrant father and a mother caregiver, (b) children with a migrant mother and a father caregiver, and (c) children with a migrant parent (mother or father) and an “other” caregiver. Hypothesis 2 expects children in the second of these groups to be especially vulnerable to emotional and conduct disorders.

#### Emotional symptoms

The proportion of children classified as having an emotional disorder on caregiver ratings ranged from 11% in Thailand to 30% in Indonesia. Basic models showed variation across the study countries (Table 4, Model C for each country).

Migration/caregiver status was sometimes significantly associated with predicted emotional disorders but not always in the same direction. Children in father-migrant/mother-caregiver households in Indonesia (*e*^*β*^ = 2.16;*β* = .77) and in Vietnam (*e*^*β*^ = 1.53;*β* = .43) had a greater odds of experiencing an emotional disorder compared to children living with both parents. In contrast, Filipino children in father-migrant/mother-caregiver households had lower odds of experiencing an emotional problem compared to those in nonmigrant households (*e*^*β*^ = 0.69;*β* = −.38). Further, in Vietnam it was children in mother-migrant/father-caregiver households who were less likely than those living with both parents to experience an emotional disorder (*e*^*β*^ = 0.52;*β* = −.65), whereas in Indonesia the association was in the opposite direction, although the results did not quite reach significance (*p* > .10). In Thailand, no significant differences were found between children living with both parents and those in transnational households.

Potential confounders, noted in previous research, were included in the extended models (Table 4, Model D for each country). The results show that, in several cases, the observed associations between migration/caregiver status and child emotional health remained significant and in the same direction once these additional variables had been accounted for. For Indonesia the odds increased, with emotional disorders more likely among children in father-migrant/mother-caregiver households compared to those living with both parents (*e*^*β*^ = 2.60;*β* = .96). In Vietnam, the equivalent estimates declined slightly and only approached significance (*p* < .10), but remained in the same direction. Vietnam is the only country where children appear to derive an emotional advantage from being left behind in the care of their fathers, with children in mother-migrant/father-caregiver households having lower odds of experiencing an emotional disorder compared to children in nonmigrant households (*e*^*β*^ = 0.51;*β* = −.67). Once selected individual and household characteristics were accounted for in the Philippines sample, there were no remaining significant differences among Filipino children living in different types of household.

#### Conduct problems

The proportion of children classified as having a conduct disorder ranged from 9% in Vietnam to 27% in Thailand. In the basic model, associations between the migration/caregiver status of children's households and reported conduct disorders again varied across the four study countries, but not in the same way as for emotional disorders (Table 5, Model C for each country). Once child age and gender were accounted for, there were no significant differences in reported conduct problems between Indonesian or Vietnamese children living in transnational households and those living with both parents.

For Thailand and the Philippines, migration/caregiver status was sometimes significantly related to child conduct disorders, but in different directions. Thai children in father-migrant/mother-caregiver households had greater odds of being reported with a conduct disorder, compared to children living with both parents (*e*^*β*^ = 1.42;*β* = .35). Filipino children in transnational households, on the other hand, appear to be *less* likely to exhibit problematic conduct, despite popular worries to the contrary. Children in father-migrant/mother-caregiver households had lower odds (*e*^*β*^ = 0.66;*β* = −.41) of having a conduct disorder whereas those in mother-migrant/father-caregiver households had even lower odds (*e*^*β*^ = 0.38;*β* = −.95); both comparisons are with children living with both parents. This result was modified slightly when other variables were added to the model.

The extended models showed that some significant associations between migration/care- giver status and child conduct disorder remained after controlling for potential confounders (Table 5, Model D for each country). Conduct disorders were more likely for Thai children in father-migrant/mother-caregiver households compared to those in nonmigrant households (*e*^*β*^ = 1.48;*β* = .39). For the Philippines, relationships remained in the opposite direction. Filipino children in mother-migrant/father-caregiver households had lower odds of having a conduct disorder compared to children living with two parents (*e*^*β*^ = 0.40;*β* = −.92). Children in father-migrant/mother-caregiver households also had lower odds (*e*^*β*^ = 0.71;*β* = −.35), although this falls just short of significance (*p* < .10).

Overall, the results present a complex picture in which psychological distress is significantly associated with transnational family arrangements for some children in some countries, but not all. Children in transnational families with “other” caregivers are not significantly different from children living with both parents in any of our models, although this finding may be compromised by small numbers. Nevertheless, there is no evidence in any of the country samples that children of migrant mothers in the care of their fathers have relatively poorer psychological well-being than children of migrant fathers in the care of their mothers, when benchmarked against children in nonmigrant households. Hypothesis 2 is, therefore, not supported. Indeed the most striking finding is that children of migrant *fathers* in the care of their mothers are more likely to suffer emotional disorders in Indonesia and conduct disorders in Thailand, compared to children living with both parents. The psychological well-being of children in transnational households in the Philippines is either better than or not significantly different from that of children in nonmigrant households, at least with respect to emotional and conduct disorders.

In general, the associations between child age and gender were in the expected directions, when significant. For example, Table 5 indicates that young children aged 3, 4, and 5 are much more likely to exhibit conduct problems than older children for all four study countries. Child age is less consistently an important factor in predicting emotional problems (Table 4) with differences only found for young children in Indonesia (*e*^*β*^ = 0.62;*β* = −.47) The effects of child gender are also more consistently significant for conduct problems, with girls less likely to exhibit conduct problems across all countries except the Philippines. Girls are significantly more likely to have emotional problems in Vietnam and the Philippines only.

Among potential confounders, those significantly associated with child psychological well-being also vary across the study countries, with one notable exception. The relationship between caregiver mental health status and both emotional symptoms and conduct problems is consistent across all models and countries. Children whose caregivers have poor mental health are between 68% (Vietnam) and 300% (Indonesia) more likely to be classified as having an emotional disorder, and between 61% (Indonesia) and 104% (the Philippines and Thailand) more likely to be classified as having a conduct disorder, compared to other children. This may be due to caregivers with poor mental health themselves overreporting psychological problems for the children in their care, as noted elsewhere (Fuhr & De Silva, 2008), although it could also be that these caregivers are struggling to cope with difficult children. Our data do not allow us to determine the direction of causation and do not contain more detailed information about caregiver mental health, but the consistency of the finding suggests an important avenue for future research.

## Discussion

If transnational labor migration is considered a family livelihood strategy that balances economic improvement against family separation, then one potential “cost” is a negative impact of separation from a parent on the psychological well-being of children left behind. This is the first study to measure psychological well-being during the period of separation for children in different types of transnational household and, comparatively, across more than one country in Southeast Asia. Caregiver-reported scores from the SDQ were used to test two hypotheses, neither of which was supported by the findings. Some previous studies led to the expectation that the psychological well-being of children separated from migrant mothers might be especially compromised, but this does not appear to be the case. Rather, in Indonesia it is children of migrant fathers who are most likely to suffer emotional disorders. The same was found for Thailand in relation to conduct disorders, although the sample does not include children of migrant mothers, thus disallowing comparison by gender of migrant parent for the Thai country sample.

The results for the Philippines are of particular interest because this is a country where transnational labor migration has been long established, the government has been most active in protecting the rights of its transnational migrants, and civil society provides more supports for those left behind. We found no evidence of poorer psychological well-being among Filipino children in transnational households compared to children in nonmigrant households. On the contrary, the results indicate that children in both father-migrant/mother-caregiver and mother-migrant/father-caregiver households are less likely to have conduct disorders and are no more likely to have emotional disorders than children living with both parents. This supports the conclusions of other recent research where Filipino children in transnational families were found to be less anxious and less lonely than their counterparts in nonmigrant families, contrary to popular perception (Asis, 2006). Perhaps the normalization of transnational families, especially in the high out-migration areas from which our sample was drawn, is protective for the psychological well-being of children with migrant parents, even when the mother is absent. Social norms are important mediators of how parent–child separation is understood. Suárez-Orozco and colleagues (2002), for example, point out that there is no stigma to child fostering in communities where it is widely practiced, and it may be that separation from a migrant parent is less traumatic when the experience is shared by neighboring children. It is also possible that modern communications, such as computer and mobile phones, play a role in keeping the absent parent “virtually present.” The Philippines is the largest source of overseas foreign workers in the region, with annual deployments more than double those from Indonesia. Such contextual factors appear to be uniquely protective in the Philippines but require further research to establish their relationship to the psychological well-being of children with a migrant parent.

Much of the prior work investigating the impact of parental migration on left-behind children in Southeast Asia has been conducted in the Philippines. This study cautions against overgeneralization because it suggests that findings for Filipino children may not be applicable across the region. We found that poorer psychological well-being is associated with transnational family arrangements for some children in each of the other study countries. Indonesian children of migrant fathers left behind in the care of their mother appear to be at greatest risk of emotional, but not conduct, disorders when compared with their peers living with both parents. Future research should examine the contextual factors that might explain this finding, including cultural norms relating to the role of women in society and reconfigurations of family life following the departure of a husband and father. For Vietnam, too, there is some evidence of an emotional cost for the children of migrant fathers, which might also be related to patriarchal gender ideologies in that country. Only for Thailand is there evidence of a relationship between migrant fathers and conduct disorders for children left in the care of their mothers. The different social and cultural contexts of transnational migration from Thailand are highlighted by the absence of migrant mothers in the sample. Transnational migration of parents is a less common occurrence in Thailand than in the Philippines or Indonesia, and in the vast majority of cases involves a father going overseas leaving children behind. Why Thai children left in the care of their mothers should be at greater risk of conduct disorders is unclear, and further research into the relationship between left-behind mothers and their children is needed. It is likely, nonetheless, that contextual factors also play a part. Comparative research for Thailand and the United States using the CBCL found that Thai respondents were more likely to include less serious aggressive child behaviors with more serious destructive child behaviors (Weisz, Weiss, Suwanlert, & Chaiyasit, 2003). The authors argue that any type of aggression is possibly considered over the threshold of social acceptability within the Thai context. The current finding about elevated conduct problems among Thai children may lend further support to this argument.

The prevalence of internalizing and externalizing disorders is known to vary based on the gender of the child across diverse cultural contexts (Crinjnen et al., 1997). The findings from this study provide some further support for the relationship between gender and externalizing disorders, whereas the support for universality of gender and internalizing disorders is less consistent. Further research should consider examining the relationship between internalizing disorders and child gender within the Asian context. It is also possible that psychological well-being and child gender may be related to gender of migrant parents. As we have established some evidence of culturally contextualized patterns of psychological well-being in this exploratory study, an important next step would be to examine more nuanced factors such as these. The previous literature on the relationship between child psychological well-being and child age is less equivocal about expected relationships, although in general early childhood is more associated with externalizing behavior problems compared to adolescence (Crinjnen et al., 1997; Egger & Angold, 2006). The current study provides further support for prior research and adds some additional information about possible differences between preschool children compared to those in middle childhood.

Household wealth, as measured by the wealth index, is also an important factor associated with emotional well-being in three of the study countries, although the relationship is not significant in the analysis for Thailand. In comparison to children living in the poorest third of households, those living in medium- or relatively high-wealth households in the Philippines and in relatively high-wealth households in Indonesia are less likely to suffer emotional problems. Wealth thus appears to be a protective factor for children, as might be expected given previous findings on socioeconomic status and mental health (Bradley & Corwyn, 2002; Hackett et al., 1999; Prior et al., 2005). In Vietnam, however, our results suggest that the opposite is the case, with children in wealthier households having poorer emotional outcomes compared to those in less wealthy households. Vietnam was chosen for inclusion in the CHAMPSEA project as a relatively new entrant to international labor migration compared to the Philippines and Indonesia. Research from the 1990s in the Philippines described the negative effects of new-found migrant wealth on child psychological well-being, in particular the problem of the “spoiled” child (Battistella & Conaco, 1998). These findings were not replicated in the more recent SMC study of migrant children in the Philippines (ECMI-CBCP/AOS–Manila, SMC, & OWWA, 2004), providing some support for the observation that, as international out-migration becomes more normative within communities, certain child behavioral problems may decrease. This provides one possible explanation for the conflicting finding for wealth in the Vietnamese context, where international out-migration is less prevalent than it is in the Philippines. Interestingly, however, wealth does not appear to be generally associated with conduct problems for the children in our samples. Only in Thailand, and then only for children in the wealthiest group of households compared to those in the poorest group, is the association significant and protective, with these children 53% less likely to exhibit conduct problems compared to their nonmigrant peers, all else being equal.

One of the strengths of this study is that the mental health of caregivers (who are also the reporters for child psychological well-being) is accounted for in the extended multivariate models. Caregiver mental health status is a consistently important predictor of emotional and conduct disorders for children in all four countries. The CHAMPSEA baseline survey did not collect detailed information about caregiver mental health beyond the WHO SRQ–20, which limits the investigation of this important influence. The current study suggests that for adverse conduct outcomes, the relative effect sizes for caregiver mental health may be somewhat larger than those for household migration/caregiver status and likewise considerably larger for adverse emotional outcomes. Although the SRQ–20 does not provide subscale scores for different psychological problems, the link between caregiver mental health and child emotional problems provides some further support for research on the strong relationship between maternal and child internalizing disorders (S. H. Goodman & Tully, 2006). Investigation of the nature of this association and related family processes must await future research to ascertain whether and in what respects caregiver depression is itself an outcome of parental absence.

This study has demonstrated differences in psychological well-being between different groups of children in four countries in Southeast Asia. Interpretation of the pattern of associations found raises a number of methodological issues, and several limitations of the study must be acknowledged. First, it must be emphasized that the sampling strategy introduced potential biases that circumscribe the interpretation of the findings. In particular, the necessarily nonrepresentative nature of the samples precludes generalization beyond the areas sampled. Moreover, in focusing on areas of high international out-migration, the samples miss children of labor migrants living in areas of lower out-migration who might suffer more from the migration of a parent. For example, children living in transnational households in areas where rates of international out-migration are lower are possibly at special risk of poor psychological well-being because of the absence of community support and peers living in similar households. The study countries were chosen to reflect a variety of policy contexts that may influence the patterns and effects of parental migration. Of particular relevance is the consideration that the presence of strong civil society support organizations, which have developed partly in response to policy efforts, may be important contributors to the observed outcomes in the Philippines. Replicated studies with other samples are necessary to confirm (or revise) the effect sizes for emotional and conduct disorders shown in Tables 4 and 5, to gain insight into the generalizability of the findings reported here, and to determine if any important unidentified confounders were excluded from the study design.

Within the context of this study, a major issue challenging the interpretation of the findings is the comparability of caregiver ratings. Are fathers as reliable as mothers in rating their child's behavior, for example? SDQ scores used in this study are based on the ratings of the principal caregiver of the target child. For children in nonmigrant households and transnational households with an absent father, this is typically the child's mother, but in transnational households with an absent mother, the caregiver may be the father, a grandparent, another relative, or even a family friend. Although Palmieri and Smith (2007) confirmed the structural validity of the SDQ in a sample of custodial grandparents in the United States, other research questions the comparability of ratings by caregivers with different relationships to the child. For example, Davé et al. (2008), in their study of SDQ scores reported by 248 parent dyads, found that fathers in the United Kingdom reported higher mean scores than mothers for externalizing behaviors, including conduct problems, and more abnormal behaviors. If such differences apply to the parental ratings used in this study, then comparisons of mother-rated and father-rated reports for different groups of children may be problematic. On the other hand, as an explanation of the differences between parental scores, Davé et al. noted that mothers are more often the principal caregivers and spend more time with their children than fathers, possibly desensitizing them to their children's problem behavior. In the present study, SDQ scores are reported by either the mother or the father (not parent dyads), with both fathers and mothers being principal caregivers of the child they are assessing. Insofar as the interactions with their child are based on a similar relationship (that of principal caregiver), their ratings may be more comparable than those of two parents of the same child, only one of whom is the child's principal caregiver.

A more important limitation concerns the use of SDQ ratings to identify cases of possible emotional and conduct disorder. This study followed other research conducted in Asia by adopting cutoffs developed for U.K. samples, as did Fuhr and De Silva (2008) for the Total Difficulties score in their study of children in Vietnam. Consequently, a substantial percentage of children (up to 30% for emotional symptoms in Indonesia) were classified in the abnormal category. This is comparable to proportions found in Pakistan (Samad et al., 2005). Nevertheless, we cannot determine whether there is a high prevalence of psychological disorders among children in our samples or whether cutoffs require adjustment to avoid false positives. Evidence from other studies is equivocal. Du and colleagues (2008), for example, confirmed U.K. cutoffs for the Emotional Symptoms and Conduct Problems subscales for a sample of Chinese children, whereas Matsuishi and colleagues (2008) adjusted U.K. cutoffs upward for the parent-rated Conduct Problems subscale based on a community sample of Japanese children. Resolution of this issue awaits normative data for the study countries.

The cross-sectional data allowed the measurement of the psychological well-being of children in different types of transnational households during a period when they had been separated from their migrant parent for at least 6 months, and the benchmarking of the results against those for children in nonmigrant households in the same communities. Migration is always selective and, although differences in socioeconomic status at the time of the interview were accounted for by including a wealth index, the relative wealth of transnational households prior to parental migration is unknown. Further, different dimensions of selection may be important in different countries. In Vietnam, for example, some communes offer loans to facilitate migration, which make it possible for less well-off parents to take up employment overseas. In the other study countries, the costs of migration may prevent poorer households from considering transnational migration as a viable livelihood strategy. Where migrants are drawn from relatively better-off households, children in transnational households may always have been less vulnerable to psychological disorders than their peers in nonmigrant households. Longitudinal data sets, which include measures of psychological well-being both before and after a parent migrates, are required to better address such problems of selectivity.

Transnational family arrangements now affect millions of children worldwide. This study suggests that some of these children may suffer psychological distress as a result of separation from a parent. In Southeast Asia, concern has focused particularly on the children of migrant mothers, who are popularly seen as being most at risk for negative impacts. It is important not to underestimate the vulnerabilities of such children over a whole range of outcomes, including physical health. Nevertheless, the results reported above provide evidence that it is children of migrant fathers left in the care of their mother in Indonesia and Thailand who are most likely to experience emotional or conduct disorders during the period of separation. This finding is not replicated for the Philippines and Vietnam. Explanation awaits further studies investigating the impacts of contact with migrant parents, gendered expectations of care, and intimate relations within both transnational and nonmigrant households on the psychological well-being of children. This study has revealed a complex picture of significant associations between parental migration and psychological outcomes for some groups of children younger than age 12, after accounting for potential confounders. The differences between the study countries are notable, suggesting that contextual factors are important to outcomes for left-behind children. Future work, as Bohr and Tse (2009) remarked, must continue to seek a balance between universal frameworks and culturally contextualized understandings.

## Note

The authors are grateful to the Wellcome Trust, U.K., for providing funding for this project (GR079946/B/06/Z and GR079946/Z/06Z). The research would not have been possible without the dedication and support of colleagues at the National University of Singapore, Scalabrini Migration Center (Philippines), Center for Population and Policy Studies, Gadjah Mada University (Indonesia), Institute for Population and Social Research, Mahidol University (Thailand), and Asia-Pacific Economic Center (Vietnam). We would also like to thank the participants in our four study countries who gave up their time to answer our questions.

## References

[b1] Adams RH, Page J (2005). Do international migration and remittances reduce poverty in developing countries?. World Development.

[b2] Ainsworth MDS, Blehar MC, Waters E, Wall S (1978). Patterns of attachment: A psychological study of the strange situation.

[b3] Amato PR, Cheadle J (2005). The long reach of divorce: Divorce and child well-being across three generations. Journal of Marriage and Family.

[b4] Asis M (2006). Living with migration: Experiences of children left-behind in the Philippines. Asian Population Studies.

[b5] Asis M, Huang S, Yeoh BSA (2004). When the light of the home is abroad: Unskilled female migration and the Filipino family. Singapore Journal of Tropical Geography.

[b6] Battistella G, Conaco CG (1998). The impact of labour migration on children left behind: A study of elementary school children in the Philippines. Sojourn.

[b7] Beiser M, Hou F, Hyman I, Tousignant M (2002). Poverty, family process, and the mental health of immigrant children in Canada. American Journal of Public Health.

[b8] Berlin LJ, Ziv Y, Amaya-Jackson L, Greenberg MT (2007). Enhancing early attachments: Theory, research, intervention, and policy.

[b9] Bernhard JK, Landolt P, Goldring L (2005). Transnational, multi-local motherhood: Experiences of separation and reunification among Latin American families in Canada.

[b10] Bohr Y, Tse C (2009). Satellite babies in transnational families: A study of parents' decisions to separate from their infants. Infant Mental Health Journal.

[b11] Bowlby J (1958). The nature of the child's tie to the mother. International Journal of Psycho-Analysis.

[b12] Bradley RH, Corwyn RF (2002). Socioeconomic status and child development. Annual Review of Psychology.

[b13] Byass P, Berhane Y, Emmelin A, Kebede D, Andersson T, Hogberg U, Wall S (2002). The role of demographic surveillance systems (DSS) in assessing the health of communities: An example from rural Ethiopia. Public Health.

[b14] Crinjnen AAM, Achenbach TM, Verhulst FC (1997). Comparisons of problems reported by parents of children in 12 cultures: Total problems, externalizing, and internalizing. Journal of American Academy of Child and Adolescent Psychiatry.

[b15] Davé S, Nazareth I, Senior R, Sherr L (2008). A comparison of father and mother report of child behaviour on the Strengths and Difficulties Questionnaire. Child Psychiatry and Human Development.

[b16] De Silva MJ, Huttly SR, Harpam T, Kenward MG (2007). Social capital and mental health: A comparative analysis of four low-income countries. Social Science and Medicine.

[b17] Du Y, Kou J, Coghill D (2008). The validity, reliability, and normative scores of the parent, teacher, and self-report versions of the Strengths and Difficulties Questionnaire in China. Child and Adolescent Psychiatry and Mental Health.

[b18] Egger HL, Angold A (2006). Common emotional and behavioral disorders in preschool children: Presentation, nosology, and epidemiology. Journal of Child Psychology and Psychiatry.

[b19] Ehrenreich B, Hochschild A, Ehrenreich B, Hochschild A, Russell A (2002). Introduction. Global women:Nannies, maids, and sex workers in the new economy.

[b20] Elmhirst R (2007). Tigers and gangsters: Masculinities and feminised migration in Indonesia. Population, Space and Place.

[b21] Episcopal Commission for the Pastoral Care of Migrants and Itinerant People—CBCP/Apostleship of the Sea–Manila, Scalabrini Migration Centre & Overseas Workers Welfare Administration [ECMI-CBCP/AOS–Manila, SMC, & OWWA] (2004). Hearts apart: Migration in the eyes of Filipino children.

[b22] Falicov CJ (2007). Working with transnational immigrants: Expanding meanings of family, community, and culture. Family Process.

[b23] Fuhr DC, De Silva MJ (2008). Physical long-term health problems and mental comorbidity: Evidence from Vietnam. Archives of Disease in Childhood.

[b24] Goodman R (1997). The Strengths and Difficulties Questionnaire: A research note. Journal of Child Psychology and Psychiatry.

[b25] Goodman R, Renfrew D, Mullick M (2000). Predicting type of psychiatric disorder from Strengths and Difficulties Questionnaire (SDQ) scores in child mental health clinics in London and Dhaka. European Child and Adolescent Psychiatry.

[b26] Goodman SH, Tully EC, Keyes CLM, Goodman SH (2006). Depression in women who are mothers: An integrative model of risk for the development of psychopathology in their sons and daughters. Women and depression: A handbook for the social, behavioral, and biomedical sciences.

[b27] Grossman KE, Grossman K, Water E (2005). Attachment from infancy to adulthood: The major longitudinal studies.

[b28] Hackett R, Hackett L, Bhakta P, Gowers S (1999). The prevalence and associations of psychiatric disorder in children in Kerala, South India. Journal of Child Psychology and Psychiatry.

[b29] Hadi A (1999). Overseas migration and the well-being of those left behind in rural communities of Bangladesh. Asia–Pacific Population Journal.

[b30] Hondagneu-Sotelo P (1994). Gendered transitions: Mexican experiences of immigration.

[b31] Hugo G (2002). Effects of international migration on the family in Indonesia. Asian and Pacific Migration Journal.

[b32] Hunt SM, Bhopal R (2004). Self report in clinical and epidemiological studies with non-English speakers: The challenge of language and culture. Journal of Epidemiology and Community Health.

[b33] Huurre T, Junkkari H, Aro H (2006). Long-term psychosocial effects of parental divorce: A follow-up study from adolescence to adulthood. European Archives of Psychiatry and Clinical Neuroscience.

[b34] Jones H, Kittisuksathit S (2003). International labour migration and quality of life: Findings from rural Thailand. International Journal of Population Geography.

[b35] Lee A, Hankin BJ (2009). Insecure attachment, dysfunctional attitudes, and low self-esteem predicting prospective symptoms of depression and anxiety during adolescence. Journal of Clinical Child and Adolescent Psychology.

[b36] Leinbach TR, Watkins JF (1998). Remittances and circulation behaviour in the livelihood process: Transmigrant families in south Sumatra, Indonesia. Economic Geography.

[b37] Levitt P (2001). The transnational villagers.

[b38] Matsuishi T, Nagano M, Araki Y, Tanaka Y, Iwasaki M, Yamashita Y, Nagamitsu S, Iizuka C, Ohya T, Shibuya K, Hara M, Matsuda K, Tsuda A, Kukuma T (2008). Scale properties of the Japanese version of the Strengths and Difficulties Questionnaire (SDQ): A study of infant and school children in community samples. Brain and Development.

[b39] McKay D (2007). “Sending dollars shows feeling”—Emotions and economies in Filipino migration. Mobilities.

[b40] Morgan C, Kirkbride J, Leff J, Craig T, Hutchinson G, McKenzie K, Morgan K, Dazzan P, Doody GA, Jones P, Murray R, Fearon P (2007). Parental separation, loss, and psychosis in different ethnic groups: A case control study. Psychological Medicine.

[b41] Mullick MSI, Goodman R (2001). Questionnaire screening for mental health problems in Bangladeshi children: A preliminary study. Social Psychiatry and Psychiatric Epidemiology.

[b42] Orellana MF, Thorne B, Chee A, Lam WSE (2001). Transnational childhoods: The participation of children in processes of family migration. Social Problems.

[b43] Palmieri PA, Smith GC (2007). Examining the structural validity of the Strengths and Difficulties Questionnaire (SDQ) in a U.S. sample of custodial grandmothers. Psychological Assessment.

[b44] Parreñas RS (2001). Mothering from a distance: Emotions, gender, and inter-generational relationships in Filipino transnational families. Feminist Studies.

[b45] Parreñas RS, Ehrenreich B, Hochschild A, Russell A (2003). The care crisis in the Philippines: Children and transnational families in the new global economy. Global women:Nannies, maids, and sex workers in the new economy.

[b46] Parreñas RS (2005). Children of global migration.

[b47] Parreñas RS (2008). Transnational fathering: Gendered conflicts, distant disciplining, and emotional gaps. Journal of Ethnic and Migration Studies.

[b48] Prior M, Virasinghe S, Smart D (2005). Behavioural problems in Sri Lankan school children: Associations with socio-economic status, age, gender, academic progress, ethnicity, and religion. Social Psychiatry and Psychiatric Epidemiology.

[b49] Samad L, Hollis C, Prince M, Goodman R (2005). Child and adolescent psychopathology in a developing country: Testing the validity of the Strengths and Difficulties Questionnaire (Urdu version). International Journal of Methods in Psychiatric Research.

[b50] Schmalzbauer L (2004). Searching for wages and mothering from afar: The case of Honduran transnational families. Journal of Marriage and Family.

[b51] Smith A, Lalonde RN, Johnson S (2004). Serial migration and its implications for the parent–child relationship: A retrospective analysis of the experiences of the children of Caribbean immigrants. Cultural Diversity and Ethnic Minority Psychology.

[b52] Smith-Estelle A, Gruskin S (2003). Vulnerability to HIV/STIs among rural women from migrant communities in Nepal: A health and human rights framework. Reproductive Health Matters.

[b53] Stark O, Bloom DE (1985). The new economics of labor migration. American Economic Review.

[b54] Suárez-Orozco C, Todorova ILG, Louie J (2002). Making up for lost time: The experience of separation and reunification among immigrant families. Family Process.

[b55] Svasek M (2008). Who cares? Families and feelings in movement. Journal of Intercultural Studies.

[b56] Syed EU, Hussein SA, Mah S (2007). Screening for emotional and behavioural problems amongst 5–11 year old children in Karachi, Pakistan. Social Psychiatry and Psychiatric Epidemiology.

[b57] Toyota M, Yeoh BSA, Nguyen L (2007). Editorial introduction: Bringing the “left-behind” back into view in Asia: A framework for understanding the “migration-left behind nexus”. Population, Space and Place.

[b58] Tuan T, Harpham T, Huong NT (2004). Validity and reliability of the self-reporting questionnaire: 20 items in Vietnam. Hong Kong Journal of Psychiatry.

[b59] Vetrovec S (2004). Migrant transnationalism and modes of transformation. International Migration Review.

[b60] Weisz JR, Weiss B, Suwanlert S, Chaiyasit W (2003). Syndromal structure of psychopathology in children of Thailand and the United States. Journal of Consulting and Clinical Psychology.

[b61] Wilson I, Huttly SRA, Fenn B (2006). A case study of sample design for longitudinal research: Young Lives. International Journal of Social Research Methodology.

[b62] Woerner W, Fleitlich-Bilyk B, Martinussen R, Fletcher J, Cucchiaro G, Dalgalarrondo P, Lui M, Tannock R (2004). The Strengths and Difficulties Questionnaire overseas: Evaluations and applications of the SDQ beyond Europe. European Child and Adolescent Psychiatry.

